# Protein structure of the venom in nine species of snake: from bio-compounds to possible healing agents

**DOI:** 10.1590/1414-431X20199001

**Published:** 2020-01-13

**Authors:** R.T. Cristina, R. Kocsis, C. Tulcan, E. Alexa, O.M. Boldura, C.I. Hulea, E. Dumitrescu, I. Radulov, F. Muselin

**Affiliations:** 1 Department of Pharmacology and Pharmacy, Faculty of Veterinary Medicine, Banat's University of Agricultural Sciences and Veterinary Medicine “King Michael I of Romania” from Timişoara, Timişoara, Romania; 2 Department of Biochemistry, Faculty of Veterinary Medicine, Banat's University of Agricultural Sciences and Veterinary Medicine “King Michael I of Romania” from Timişoara, Timişoara, Romania; 3 Department of Chemistry, Faculty of Agriculture, Banat's University of Agricultural Sciences and Veterinary Medicine “King Michael I of Romania” from Timişoara, Timişoara, Romania; 4 Department of Toxicology, Faculty of Veterinary Medicine, Banat's University of Agriculture and Veterinary Medicine “King Michael I of Romania” from Timişoara, Timişoara, Romania

**Keywords:** Bio-compounds, Protein LabChip, Venomics, Vipera aspis, V. xantina palestinae

## Abstract

Due to its various structures in bio-compounds, snake venom is the indisputable result of evolutionary stages of molecules with an increasingly complex structure, high specificity, and of great importance for medicine because of their potential. The present study proposed an underpinning examination of venom composition from nine species of venomous snakes using a useful and replicable methodology. The objective was the extension of the evaluation of protein fractions in the field up to 230 kDa to permit possible identification of some fractions that are insufficiently studied. The gel capillary electrophoresis method on the chip was performed using an Agilent 2100 bioassay with the 80 and 230-LabChip Protein kits. Interpretation of electrophoresis was performed using the Protein 2100 expert (Agilent) test software as follows: a) Protein 80 (peak size scale): 1.60, 3.5, 6.50, 15.00, 28.00, 46.00, 63.00, 95.00 kDa; b) Protein 230 (peak size scale): 4.50, 7.00, 15.00, 28.00, 46.00, 63.00, 95.00, 150.00, 240.00 kDa. The screening revealed the presence of compounds with a molecular weight greater than 80 kDa, in the case of *Vipera aspis* and *Vipera xantina palestinae*. For *V. aspis*, a 125 kDa molecular weight pro-coagulant protein was identified, known as being involved in the reduction of plasma clotting time without any direct activity in the fibrinogen coagulation process. The samples examined on the Protein 230-LabChip electrophoresis chip can be considered as a novelty with possible uses in medicine, requiring further approaches by advanced proteomics techniques to confirm the intimate structural features and biological properties of snake venoms.

## Introduction

Due to the various structures of bio-compounds (e.g., peptides, toxins, enzymes, up to 100 proteins, and different isoforms), snake venoms are the indisputable result of evolutionary stages for molecules with an increasingly complex structure and high specificity (1–3).

This high protein amino acid abundance, generally possessing specific enzymatic and polypeptide characteristics, is often different from one snake species to another, but authors generally agree that these assemblies can be classified into several common chemical families like: phosphodiesterases (4), acetylcholinesterases (5), phospholipases (6,7), proteases (serine and metalloproteases) (8–10), disintegrins (11,12), as well as the so-called “three-finger toxins” tri-toxins: neuro-cardio-hemodyotoxins (13). Certainly, this large blend of biochemical molecules present in snake venoms makes these various and complex structures attractive to the investigation of new therapeutic resources (14,15).

Venom components are recognized as effective in the treatment of blood pathology, mainly in hemostasis and anticoagulation/coagulation processes (16–18), in hypertension, influencing angiotensin-converting enzyme inhibitors, or in renal disease (19).

Also, the disintegrins in the venom have been shown to be integrin modulators, with definite anti-tumor, metastatic, or anti-angiogenic activity (20–22). These new activities were also studied for possible treatments of arthritis and thrombosis (22).

Knowing that snake venom contains a very wide range of biological structures, studies of structure identification and biological and pharmaco-clinical activity are now of great importance. In this context, the present study proposed an examination of venom composition from nine species of venomous snakes belonging to the *Viperidae* and *Crotalinae* genus using a useful and replicable methodology.

The extension of protein fractions evaluation in the field up to 230 kDa allows the identification of fractions that are insufficiently studied so far, including both their structures and their biological effects.

## Material and Methods

### Venom collection

In all cases, animal manipulation, including snakes' harvesting, was in line with the UNC Institutional Animal Care and Use Committee approved protocols, and none of the animals were on the International Union for Conservation of Nature threatened species list.

Fresh venom samples were obtained from nine different species of snakes. The person responsible for the venom gathering was an expert in exotic pathology, the owner of a specialized exotic animals clinic, and a certified veterinarian in snake venom collection. The samples were gathered from pet snakes living in home terrariums and usually registered and treated in this clinic. For venom collection, the classical technique from the literature was used (23,24).

After sampling, the venom was air-dried and the samples stored in a crystalline state in a freezer at −80±2°C until the chemical analysis was performed (25).

### Reagents and equipment used

The reagents used were: bovine serum albumin (BSA) (Sigma Aldrich, Germany), Folin Ciocalteu reagent (Merck, Germany), Na_2_CO_3_, NaOH, Na_2_-tartrate × 2H_2_O, all analytical grades (Merck), ultrapure water (Waters Millipore, Germany).

The equipment used for sample preparation and analyses were: analytical scale Kern EG 420-3NM (Germany), Hettich Universal-320R centrifuge (Germany), IKA-4 digital Vortex centrifuge (Germany), Agilent 2100 bio-analyzer (USA), MilliQ integral 5 Pure System - Ultrapure Water Station (Germany), and Thermo Scientific 902 ultra-freezer (USA). Chromatographic analysis was performed on a Perkin Elmer - Lambda 25 spectrophotometer (USA).

### Freeze drying methodology

The working procedure included: weighing the initially crystallized venom, solubilization of crystalline venom, rapid freeze-drying, preparing the ampoules, homogenizing the final product, and final weighing. The lyophilizer used in our experiment was one Ilshin Kryptonstraat 11_6718_WR_EDE (Ilshin, The Netherlands) with the following parameters: freeze-drying: −54°C, 5 mTorr for 48 h; freezing yield was between 76.80−89.16%.

### Validation method

Validation was done by the determination of the solid substance, according to the known standardized method at 103°C. The ampoule with the sample was kept for 12 h at 103°C. The vial was then inserted into the dryer for cooling. After cooling, the vial was weighed with an accuracy of 0.0001 g. The heating operation was repeated for one hour, cooling and weighing until the results obtained on two successive weighing did not differ by more than 0.1%. The results were compared with freeze-dried venom water content in order to optimize the freeze-drying conditions. The freeze-drying yield was calculated as a percentage of the dry matter obtained by comparison with the initial amount contained therein. The samples were lyophilized and stored in the freezer at −80°C in Eppendorf tubes and sealed with paraffin foil to prevent wetting of the samples, according to WHO Guidelines (2016) for the Production, Control and Regulation of Snake Antivenom Immunoglobulins (https://www.who.int/biologicals/expert_committee/Antivenom_WHO_Guidelines_DJW_DEB_mn_cp.pdf?ua%20=%201).

### Gel capillary electrophoresis (CGE) on laser-induced fluorescence detection chip

The CGE method on chip was performed using an Agilent 2100 bioassay (Agilent Technologies, Germany) with the 80-LabChip Protein and 230-LabChip Protein kits, according to the protocol described by the manufacturer and following the methodology described by Halassy et al. (26).

Prior to electrophoresis, the samples were diluted in 30 mM Tris/HCl at pH 8.5 to a concentration of 10 mg/mL (4 μL of the diluted samples of each type of venom were mixed with 2 μL of buffer containing a reducing agent, in our case, β-mercapto-ethanol). The supplied samples and kit scale were then denatured for 5 min at 95°C and then diluted with 84 μL of sterile solution of MilliQ H_2_O. After this treatment, the samples and the scale migrated to the CGE chip and were measured immediately. Interpretation of electrophoresis was performed using the manufacturer's Protein 2100 expert (Agilent) test software for peak detection; quantity and quality of protein fractions were detected as follows: a) Protein 80 (peak size scale): 1.60, 3.5, 6.50, 15.00, 28.00, 46.00, 63.00, 95.00 kDa; and b) Protein 230 (peak size scale): 4.50, 7.00, 15.00, 28.00, 46.00, 63.00, 95.00, 150.00, 240.00 kDa.

## Results and Discussion

### Determination of protein content

**Figure 1:**
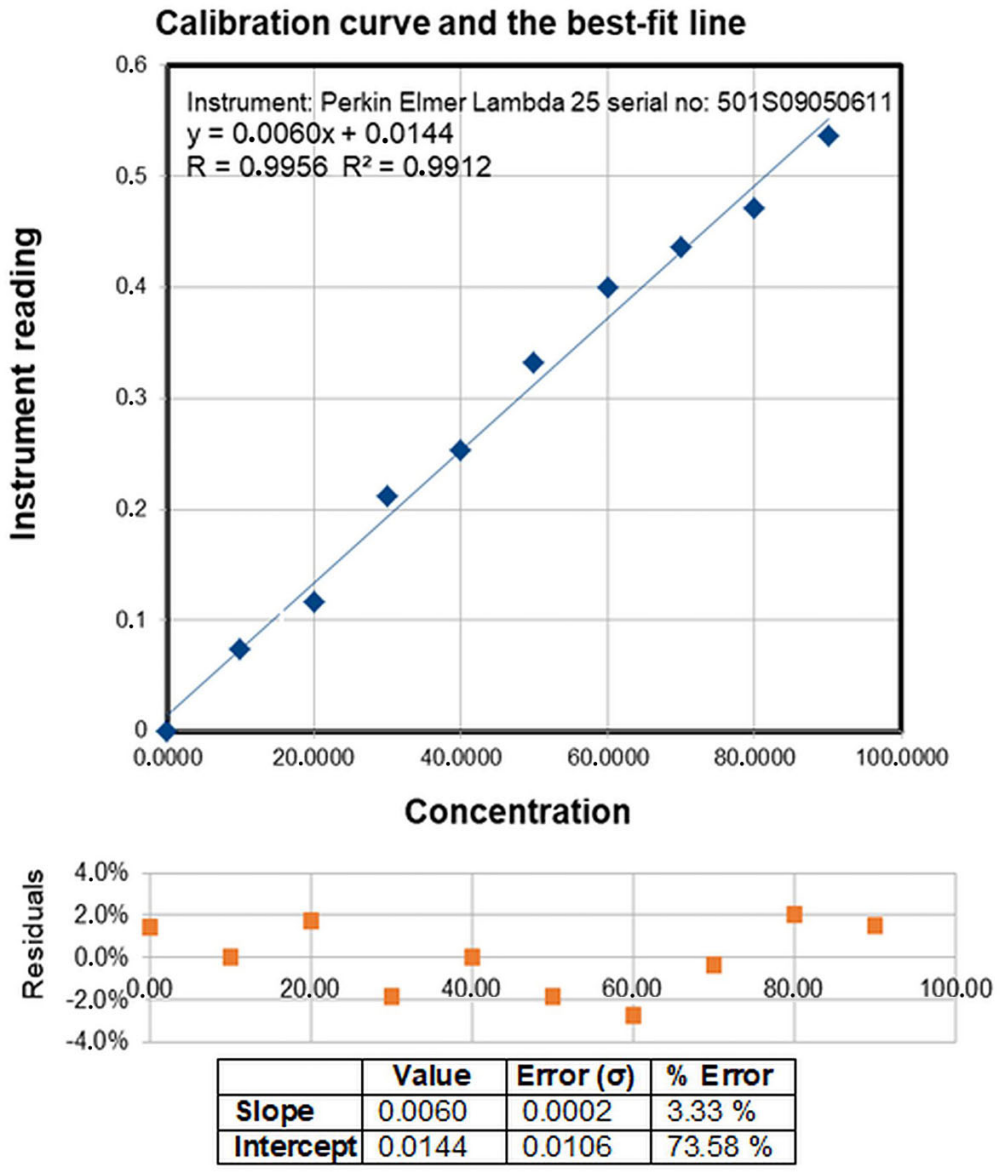
Calibration curve for total protein content in the venom.

The total protein content of the samples was determined according to the known classical methodology originally provided by Lowry in 1951, which was adapted for venom by Dutta et al. (27), the calibration curve being prepared with BSA (Figure 1). The results are reported as percentage of the protein content of the total venom mass as means±SD of three analyzes/sample (Table 1).

**Table 1:** Total protein content of freeze-dried venom samples of snake species.

Snake species	Protein content
*Crotalus horridus horridus*	92.37±0.83
*Crotalus molossus nigrescens*	92.03±0.77
*Crotalus scutulatus salvini*	91.98±0.52
*Crotalus simus tzabcan*	92.11±0.94
*Crotalus vegrandis*	91.25±0.43
*Vipera aspis*	91.24±0.29
*Vipera bitis arietans*	90.27±0.42
*Vipera bitis nasicornis*	91.52±0.38
*Vipera xantina palestinae*	92.01±0.63

Data are reported as means±SD in percent.

**Figure 2:**
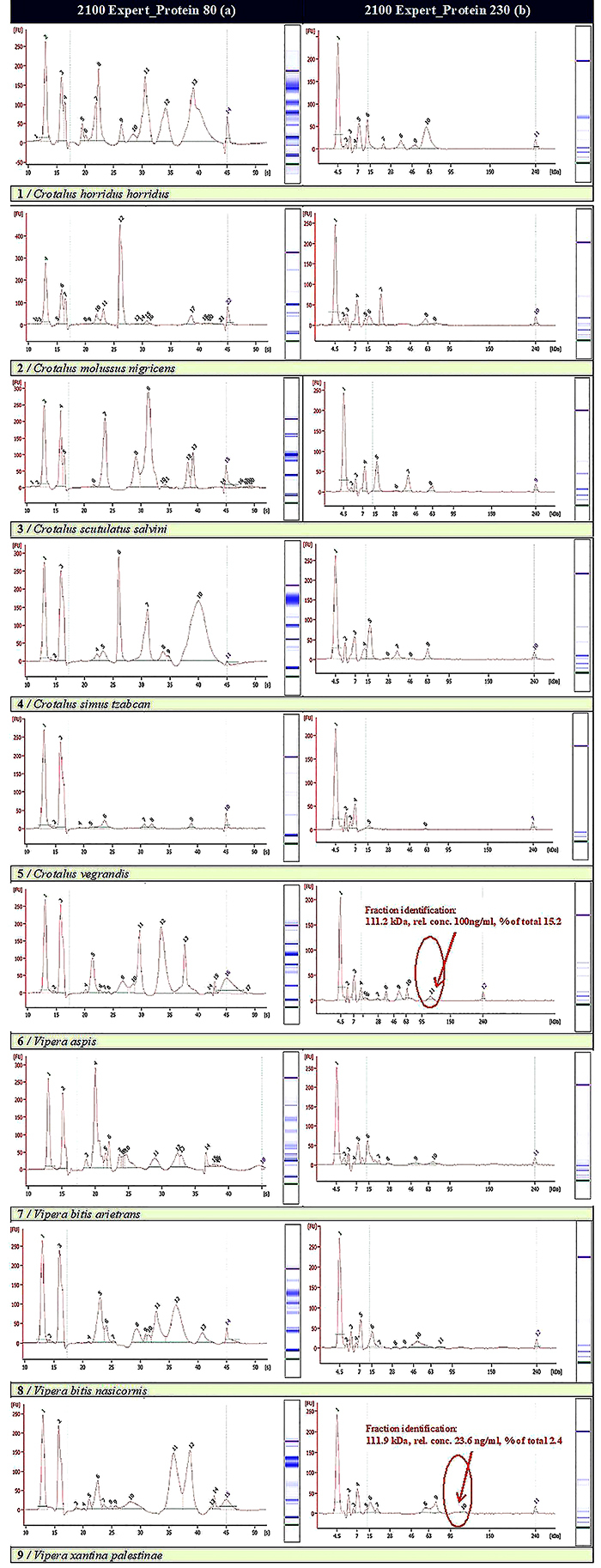
Electropherograms of lyophilized venom samples showing peak detection, and quantity and quality of detected protein fractions: **a**) Protein 80 and **b**) Protein 230, using Protein 2100 Expert Testing (Agilent).

The values of the electropherograms obtained on the lyophilized venom samples are shown in Figure 2.

Our approach can be classified into the ever-increasing number of preliminary projections for identifying the potential innovative therapeutic tools offered by snake venom. In all of these initial studies, the main objective was to identify with certainty new structures/fractions with possible health benefits (28–30).

The lowest molecular mass molecule determined was observed in interval 6–7 kDa and is related to the presence of non-PLA2 (phospholipase A2) myotoxins that play a role of decreasing the post-envenomation prey trek. We observed that the *Crotalus vegrandis* venom contained the highest fraction from the analyzed sample, traces of this compound being detected also in *Vipera bitis arietans* and *Vipera xantina palestinae*.

Undoubtedly, this large blend of biochemical molecules present in snake venoms makes these various and complex structures attractive to investigate new therapeutic resources. We have also identified short- and medium-size disintegrins in the range of 7–10 kDa. Bitistatin and crotatroxin act like disintegrins for α IIb; β 5 and α v integrins, with ligands like collagen, fibronectin, vitronectin, fibrinogen, and vitronectin, have known functions in angiogenesis, migration, and invasion processes (31,32).

These molecules are derived from snake venom metalloproteases (SVMP), phylogenetically related with ADAM (a disintegrin and metalloproteinase) and ADAMTS (a disintegrin-like and metalloproteinase with thrombospondin type-1 motifs), extracellular protease enzymes multi-domain family also present in venom. These proteins have the capacity to interact with specific integrins and inhibition of their activity has led to the discovery of potential diagnostic and therapeutic agents in oncology (32).

Phospholipase A, identified by us with the molecular mass interval between 12–17 kDa, is a Ca^2+^-dependent enzyme, which hydrolyzes 2-acyl groups from 3-sn-phosphoglycerides, with pharmacological effects such as myonecrosis, lipid membrane damage, cardiotoxicity, platelet aggregation initiation/inhibition, and post- and presynaptic neurotoxicity. We have identified the PLA2 group II A subgroup in rattle snake and viper venoms, confirming the research of Fox and Serrano (10).

Classification in subgroups is determined by the 49th position amino acid, known to have a crucial role in catalysis. Despite the fact that these enzymes exist mostly as monomers, the molecular mass variation could be the result of aggregates or complexes formed by the covalent or non-covalent interactions between PLA2 and other proteins. The effect of additional protein association is decisive in amplifying the pharmacological effect. This is the specific case of crotoxin and many other crotoxin-like neurotoxins, like “Mojave toxin” from *Crotalus scutulatus salvini* or vegrandis toxin from *Crotalus vegrandis*, which can explain the occurrence of 15–17 kDa fractions for this species (in a proportion of 27.3 and 58.3%, respectively) (10).

The Vipera toxins contain a representative of the heterodimeric PLA2 group, identified in the 13–14 kDa molecular mass interval with an occurrence of 15.4% for *Vipera xantina palestinae*; it was firstly described by Ovadia (34) and then confirmed by other authors (35–37).

In the range of 21–24 kDa, *Crotalus molossus nigrescens* registered the highest concentration (67.5%) fraction, separated and described as a proteinase E with a molecular weight of 21,390 and the following N-terminal amino acid sequence; Phe-Ala-Lys-Arg-Tyr-Val-Glx-Leu-Val-Ile-Val-Ala (4,14).

SVMPs are the most abundant toxins in viperid venoms and have evolved from ADAM family members (most likely from ADAM 7, a non-catalytic like metalloprotease, and ADAM 28, a proteolytic metalloprotease with activity on the extracellular matrix) (9,10,12,33).

These are considered multi-domain proteins and are structured in sub-classes also described by other authors (3,4,10,12,15,18–20,22,34–40) as being: P-I (25–30 kDa) with hemorrhagic and/or non-hemorrhagic action, P-IIa (30–45 kDa) with hemorrhagic action, P-IIb (30–45 kDa) inhibition of platelet aggregation, P-III (50–100 kDa) with hemorrhagic, apoptotic, and factor X activation. In P-I subclass (P-I SVMPs only), it was observed that the hemorrhagic minimal dose is greater than that described for subclass P-III.

Metalloproteinase P-II c, b1-1 was isolated and characterized from *Agkistrodon bilineatus* venom. It had no platelet aggregation activity and was determined in 8% in *Agkistrodon bilineatus* (30–40 kDa range) (38). In our case, the highest levels of Zn metalloproteinase P-III were identified in the venom of *Crotalus horridus horridus*, *Vipera aspis*, *Vipera xantina palestinae*, and at the highest level (50.4%) in the *Crotalus simus tzabcan* venom.

In the 60-kDa range, the major component (37.1%) was identified by us in *Vipera xantina* and to some extent in *Vipera aspis*, and it could be related to the class of three hemorrhagic factors with intense proteolytic activity, with casein and gelatin substrate already having been described in the literature (36–40).

A less intensive enzyme studied in snake venom is hyaluronidase, with a molecular weight that can reach 110 kDa. It has already been isolated in *Agkistrodon* venom. This appears to provide extracellular matrix fragmentation and systemic toxin diffusion into the bloodstream (38). The largest fraction we identified for hyaluronidase was of 70–80 kDa, (29.6%). Despite demonstrating the appearance of all major snake venom components in the 80-LabChip Protein domain, we also performed parallel electropherograms for the 230-LabChip Protein domain. The analysis revealed a 125-kDa molecular weight protein for *Vipera aspis* that has been described as a pro-coagulant protein. This activity was described as being involved in the reduction of plasma clotting time without any direct activity in the fibrinogen coagulation process (36,37,39,40).

Researchers are fervent in finding new protein sequences with certain biological activity, the potential of snake venoms being undoubtedly ascertained. In the last decade, different types of venomic structures were identified by recognized groups of researchers, their “small steps” in this topic being crucial for today's medical science (Supplementary Table S1).

This preliminary study represents the starting point in the developing method for isolation and characterization of large size proteins in snake venom. In our case, the evidence of compounds with a molecular weight greater than 80 kDa (111.2 and 111.9 kDa), in the case of *Vipera aspis* and *Vipera xantina palestinae*, samples examined on the Protein 230-LabChip electrophoresis chip can be considered a novelty with possible uses in medicine, requiring further approaches by advanced proteomics techniques to confirm the structural features and biological properties.

## Supplementary material

Click here to view [pdf].
